# Supplementary material for: Acceptance and compliance with micronutrient powder (MNP) among children aged 6–23 months in northern Nigeria
Source: PLOS Glob Public Health. 2022 Oct 17;2(10):e0000961. doi: 10.1371/journal.pgph.0000961 (PMC10022258; doi:10.1371/journal.pgph.0000961)
Supplement: S1 Dataset — (ZIP) [file pgph.0000961.s008.zip › S8_Dataset/Direct Observations.zip!/Direct Observations/270605_A_GU_AD_1_DO.pdf]

1.1.1 Instrument F –Direct Observation Form

<<Instrument F Direct Observation Form>>

PI Name: PragyaMathema,Stephen Kodish
Study Title: Formative research to develop an infant and young child feeding (IYCF) intervention with micronutrient powder (MNP) for children aged 6 – 23 months
PI Version/Date: Version1.0/January 24, 2015

Date: _June 27, 2015_____________Location: _ Lakumna community,GUYUK, LGA_________

Data Collector: Aisha Aliyu Dahiru_______________Participant ID: __Mud house with Neem tree in front

Main goals of this direct observation are to answer the following questions:

1. In this household, what would be the best way(s) to give nutritional supplements to young children? Why?

2. In this household, describe the food sharing that you observe throughout the meal. Are there any predictable patterns?

3. In this household, who is favored and how is this favoritism expressed during meal preparation and eating?

(TEAR OFF AT PERFORATED DOTTED LINE BELOW)

PARTICIPANT ID: small hut with low verandah__

Household Composition Table (Fill out as you do data collection)

INITIALS	SEX (M/F)	AGE (estimated)	Relationship to household	Description
M	F	26YRS	Mother	Wearing pink shirt with plaited hair.
G	F	16 MONTHS	Baby girl daughter	Wearing brown dress
N1	F	26 YRS	Neighbour	Wearing yellow wrapper
N2	F	24 YRS	Neighbor	Wearing blue wrapper and carrying baby on her back
GM	F	70YRS	Grand mother	Wearing black shirt and a head tie
N3	F	29 YRS	Neighbour	Wearing purple stripped shirt with orange wrapper
OC	M	5 years	Older son	Wearing green shirt

PARTICIPANT ID: _small hut wit low verandah________

TIME	ACTOR INITIALS	RECIPIENT INITIALS	WHAT HAPPENED	FOOD	Reactivity
8.00	M		Washing utensils		
8:05	M		Washing utensils		
8:10	M		Washing utensils		
8:15	M		Washing utensils		
8:20	M		Washing utensils		
8:25	M		Keeping the utensils in a corner in the house		
8:30	M		Pick broom and start sweeping		
8:35	M		Sweeping		
8:40	M		Sweeping		
8:45	M		Sweeping		
8:50	M		Starting fire		

TIME	ACTOR INITIALS	RECIPIENT INITIALS	WHAT HAPPENED	FOOD	Reactivity
8:55	M		Starting fire		
9:00	M		Placing pot with water on fire	water	
9:00	M	G	Carrying baby on her back		
9:05	M		Carrying baby on her back		
9:05	M		Brings out guinea corn flour from the room	Guinea corn flour	
9:10	M		Takes a portion of guinea corn flour in a small bowl and mix with water	Guinea corn flour with water	
9:15	M		Adding guinea corn mixture to boiling water on fire	Guinea corn flour with water	
9:15	M	G	Carrying baby on her back		
9:20	M		Kunu is ready to be served	Kunun dawa	
9:20			Serving kunu into flask	Kunun dawa	
9:25	M		Serving kunu into a cup and keeping it to cool down	Kunun dawa	
9:30	M	G	Feeding baby with kunu using spoon. Baby is taking the kunu without any resistance	Kunun dawa	
9:31	M	G	Feeding the baby	Kunun dawa	
9:32	M	G	Feeding the baby	Kunun dawa	
9:33	M	G	Feeding the baby	Kunun dawa	
9:34	M	G	Feeding the baby	Kunun dawa	

TIME	ACTOR INITIALS	RECIPIENT INITIALS	WHAT HAPPENED	FOOD	Reactivity
9:35	M	G	Washing the baby hands and mouth		
9:40	M	G	Bathing the baby		
9:45	M	G	Bathing the baby		
9:50	M	G	Bathing the baby		
9:55	M	G	Dressing the baby		
9:55	M	G	Combing the baby's hair		
10:00	G		Baby playing on the mat		
10:05	G		Playing on the mat		
10:10	M		Washing clothes		
10:15	M		Washing clothes		
10:20	M		Washing clothes		
10:25	M		Washing clothes		
10:30	M		Washing clothes		
10:30	G		Playing on the mat		
10:35	M		Spreading clothes to dry on the fence		
10:35	G		Playing on the mat		
10:40	G		Goes out and enters next house. The grandmother house		
10:45	G		Sitting on the mat beside the grandmother		
10:50	GM	G	Playing with the baby and tickling her belle		
10:55	GM	G	Pick up the baby and carry her on her lap		
10:55	GM	G	Playing with the baby		
11:00	GM	G	Playing with the baby		
11:00	G		Baby goes to play with other children under the shade		
11:05	G		Playing around the house with other children		

TIME	ACTOR INITIALS	RECIPIENT INITIALS	WHAT HAPPENED	FOOD	Reactivity
11:10	G		playing with other children		
11:15	G		Goes back to other and sits on her lap		
11:15	G		Sitting on mother lap playing		
11:20	M	G	Mother breastfeeding baby	Breast milk	
11:21	M	G	Mother breastfeeding baby	Breast milk	
11:22	M	G	Mother breastfeeding baby	Breast milk	
11:27	G		Playing on the mat		
11:32	G		Playing on the mat		
11:37	M		Sitting on the mat beside the baby		
11:42	N1	M	Comes in greeting the mother		
11:42	M	N1	Chatting with the neighbor		
11:47	M	N1	Chatting with the neighbor		
11:52	N2		Comes into the house, greeting mother		
11:52	M	N1,N2	Chatting with the neighbors		
11:52	G		Playing on mother’s lap		
11:57	N1	N2	Goes out chatting with the neighbors		
11:57	M	G	Playing baby on the mat		
12:02	G		Playing on the mat by herself		
12:07	M		Brings tuwo from grandmothers house	Tuwon dawa	
12:07	G		Crying that the bowl of tuwon	Tuwon	

TIME	ACTOR INITIALS	RECIPIENT INITIALS	WHAT HAPPENED	FOOD	Reactivity
			should be given to her	dawa, okro soup	
12:07	M	G	Washing baby's hand where she is sitting		
12:12	G		Eating tuwo with her hand	Tuwon masara, okro soup	
12:13	G		Eating tuwo with her hands	Tuwon masara, okro soup	
12:14	M		Joins baby to eat tuwo	Tuwon masara, okro soup	
12:15	M	G	Assisting the baby to eat but the baby refused after collecting the first two morsels. The baby insists on eating with her hands.	Tuwon masara, okro soup	
12:16	M		Eating with the baby		
12:17	G		Eating with her hand	Tuwon masara	
12:18	G		Baby is drinking water from a cup beside the mother	Water	
12:23	M	G	Mother washing baby hands and mouth		
12:23	G		Picks the bowl of tuwo and licked it		
12:23	M	G	Collect the bowl from the baby		
12:23	G		Crying and protesting because of the bowl		
12:28	M	G	Consoling the baby		

TIME	ACTOR INITIALS	RECIPIENT INITIALS	WHAT HAPPENED	FOOD	Reactivity
12:33	G		Playing on mother's lap		
12:38	M		Sitting by the door post with the baby		
12:43	G		Playing with padlock on the room door		
12:48	G		Playing with padlock and bowl beside the mother in front of the room		
12:53	G		Playing beside mother		
12:58	M	G	Teaching baby some words in local language		
1:03	G		Repeating each word that the mother speaks		
1:08	M	G	Bathing baby		
1:13	M	G	Keep baby on the mat		
1:18	G		Playing on the mat with a toy phone		
1:23	M	G	Dressing baby		
1:28	M	G	Playing with baby		
1:33	M	G	Breastfeeding the baby	Breast milk	
1:34	M	G	Breastfeeding the baby	Breast milk	
1:45	M	G	Breastfeeding the baby	Breast milk	
1:46	M	G	Breastfeeding the baby	Breast milk	
1:46	G		Baby is sleeping		
1:51	G		sleeping		
1:56	G		Sleeping		
2:01	G		Sleeping		
2:06	G		sleeping		

TIME	ACTOR INITIALS	RECIPIENT INITIALS	WHAT HAPPENED	FOOD	Reactivity
2:11	G		sleeping		
2:16	G		sleeping		
2:21	G		sleeping		
2:26	G		sleeping		
2:31	M		Sitting under the tree in front of the house		
2:36	M	N1,N2	Chatting with neighbours		
2:41	M	N1,N2	Chatting with neighbours		
2:46	N1		Breastfeeding her baby	Breast milk	
2:51	N2		Combing daughters hair		
2:56	G		Sleeping		
3:01	G		Sleeping		
3:06	M	N1,N2	Chatting with neighbours		
3:11	N3		Join mother and neighbor under the tree		
3:16	M	N1,N2,N3	Chatting with neighbor		
3:21	G		Sleeping		
3:26	G		Wakes up crying		
3:31	M	G	Pick up crying baby		
3:36	M	G	Consoling baby, sitting under the tree		
3:41	M	G	Consoling baby , sitting under the tree		
3:46	G		Playing on the mat under the tree		
3:51	M	G	Carrying baby on lap		
3:56	M	G	Breastfeeding baby	Breast milk	
4:01	M	G	Breastfeeding baby	Breast milk	
4:02	M	G	Breastfeeding baby	Breast milk	
4:03	M	G	Breastfeeding baby	Breast	

TIME	ACTOR INITIALS	RECIPIENT INITIALS	WHAT HAPPENED	FOOD	Reactivity
				milk	
4:04	M	G	Breastfeeding baby	Breast milk	
4:05	M	G	Baby playing on the ground		
4:10	G		Baby playing on the ground		
4:15	M		Goes back into the house and bring out flask from the room		
4:20	M		Serving kunu into large cup from the flask	Kunun dawa	
4:25	M		Serves a little quantity of kunu into smaller cup from the large cup	Kunun dawa	
4:25	M		Adding MNP into smaller cup and mixing it with the kunu	MNP, kunun dawa	
4:30	G		Pulling mother wrapper and waiting to be fed		
4:30	M	G	Feeding baby with the cup and keeping spoon aside	Kunun dawa	
4:30	G		Drinking the kunu with enthusiasm licking her lips and nodding sitting on the ground	Kunu dawa	
4:31	M	G	Encouraging the baby while feeding her to finish the kunu	Kunun dawa	
4:32	M	G	Feeding the baby with spoon	Kunun dawa	
4:33	M	G	Carrying baby on her life still feeding the baby	Kunun dawa	
4:34	M	G	Still feeding the baby with spoon	Kunun dawa	
4:3	G		Nodding and playing with her legs while taking the kunu. The baby drank the whole kunun that was mixed with MNP.	Kunun dawa	
4:35	M	G	Giving the baby water to drink	Water	
4:35	M	G	Washing the baby hands and mouth		
4:40	M		Washing the utensils used in feeding the baby		
4:45	M		Carrying baby on her back		
4:45	M		Starting fire to boil water	water	
4:50	M		Starting fire to boil water	Water	

TIME	ACTOR INITIALS	RECIPIENT INITIALS	WHAT HAPPENED	FOOD	Reactivity
4:55	M		Mixing millet flour into boiling water to make meal	Millet flour meal	
5:00	M		Still adding millet flour into boiling water to make meal	Millet flour	
5:05	M		Still stirring the mixture	Millet flour water	
5:10	M		Meal is ready to be served	Millet meal	
5:10	M		Serving meal into two separate bowls	Millet meal	
5:15	M		Washing the utensils used in preparing the meal		
5:20	M		Placing another pot on the fire to warm the soup. The soup was prepared earlier in the morning in our absence	Okro soup	
5:25	M		Dishing out the soup into another bowl	Okro soup	
5:30	M	G	Carrying baby on her back		
5:35	M	G	Washing the baby’s hand		
5:40	M	G	Mother is feeding the child from her bowl while the baby is sitting on her lap	Millet meal, okro soup	
5:41	M	G	Mother is feeding the baby sitting on her lap	Millet meal, okro soup	
5:42	M	G	Feeding the baby on her lap	Millet meal, okro soup	
5:43	G		Baby is eating by herself	Millet meal, okro soup	
5:44	G		Baby girl is eating by herself	Millet meal,	

TIME	ACTOR INITIALS	RECIPIENT INITIALS	WHAT HAPPENED	FOOD	Reactivity
				okro soup	
5:45	M	G	Mother is assisting the baby to eat	Millet meal, okro soup	
5:45	OC		Older son is eating in a separate bowl	Millet meal, okro soup	
5:46	OC	G	Older son trying to feed baby the morsel of food offered by her brother	Millet meal, okro soup	
5:47	OC	G	Assisting the baby girl to eat	Millet meal, okro soup	
5:48	M	G	Assisting the baby girl to eat	Millet meal, okro soup	
5:49	G		Eating meal together with mother on her laps. The baby is eating with her hands	Millet meal, okro soup	
5:50	M	G	Mother is washing the child hands		
5:50	OC		Washing hands supervised by mother		
5:55	OC	G	Older child paying with the baby girl		
6:00	M		Washing the utensils used in eating meal		

TIME	ACTOR INITIALS	RECIPIENT INITIALS	WHAT HAPPENED	FOOD	Reactivity

TIME	ACTOR INITIALS	RECIPIENT INITIALS	WHAT HAPPENED	FOOD	Reactivity

PARTICIPANT ID: __ _______

Primary Questions (Complete after you complete data collection):

From what you have seen observing THIS HOUSEHOLD, please answer the following questions in detail:

Question	Write your detailed response here
1. In this household, what would be the best way(s) to give nutritional supplements such as an MNP to young children to ensure compliance?<br><br>Why?	The best way to ensure compliance is to educate and train the mother and grandmother on MNP.
2. In this household, who is favored during meal preparation or feeding?<br><br>How is this favoritism expressed?	The parents and older child are favored during meal preparation and feeding because the food prepared was in solid state and they had separate bowls each.<br><br>The baby was fed carefully and diligently.
3. How could we promote the MNP supplement for this family that would attract their attention?<br><br>Who would they listen to?	MNP can be promoted to this household through the mother and the grandmother because the grandmother too was curious about the program, its benefits and how it will be used. I noticed that she has a say in the things that were going on in the household.<br>It can also be promoted through the community leaders because the women were chatting on how he has encouraged them to

	accept such kind of help in the past to benefit themselves and their children.
4. In this household, what would be the best ways to promote the MNP but limit how much it would be shared?<br><br>Why?	The best way to limit MNP sharing in this household is to ensure that the mother understands that is meant for the child that is 6 - 23 months only and should be given separately.<br>Also, the mother should ensure child she feed the child MNP before her meal or after and ensure the older child does not try to eat part of it.
5. What other issues and concerns did you see with this family that are important for us to consider with regard to introduction of an MNP?<br><br>Why?	The food prepared later in the day was solid and cannot be used to administer MNP.<br>The mother was in charge of feeding the child.

6. Based on your experience observing this child today, do you believe reactivity negatively impacted your ability to see the food-related behaviors of the child?<br><br>Please explain your answer in detail.	There was no reactivity during feeding because the mother said the child usually eats in the manner she ate.<br><br>The child drank her kunu and ate the solid food with ease and ate mostly with her hands.
